# Supplementary material for: Marine biofilms constitute a bank of hidden microbial diversity and functional potential
Source: Nat Commun. 2019 Jan 31;10:517. doi: 10.1038/s41467-019-08463-z (PMC6355793; doi:10.1038/s41467-019-08463-z)
Supplement: Supplementary file 1 — Supplementary Information [file 41467_2019_8463_MOESM1_ESM.pdf]

Biofilms constitute a bank of hidden microbial diversity and functional potential in the oceans

Zhang et al

Supplementary Information

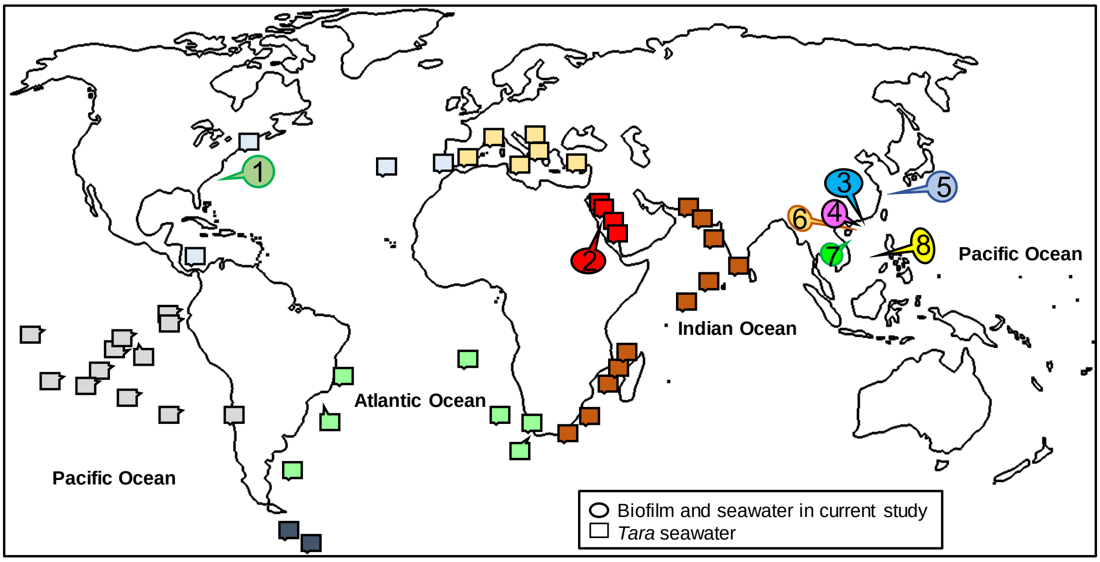

Supplementary Figure 1. Geographic distribution of the eight sampling locations of the 101 biofilms. (1) South Atlantic, (2) Red Sea, (3) Hong Kong Water, (4) Yung Shu O Bay, (5) East China Sea, (6) South China Sea 1, (7) South China Sea 2, and (8) South China Sea 3. The majority of biofilms were developed on Petri dishes and man-made panels immersed in the subtidal zone. Biofilms were sampled from Hong Kong seawater in different months during 2013-2017. During biofilm development, 24 adjacent seawater samples were collected from the locations (2), (3), (4), and (6) for comparison. Sampling locations of the 67 *Tara* seawater samples (surface seawater metagenomes downloaded for comparison with the biofilms) are also shown.

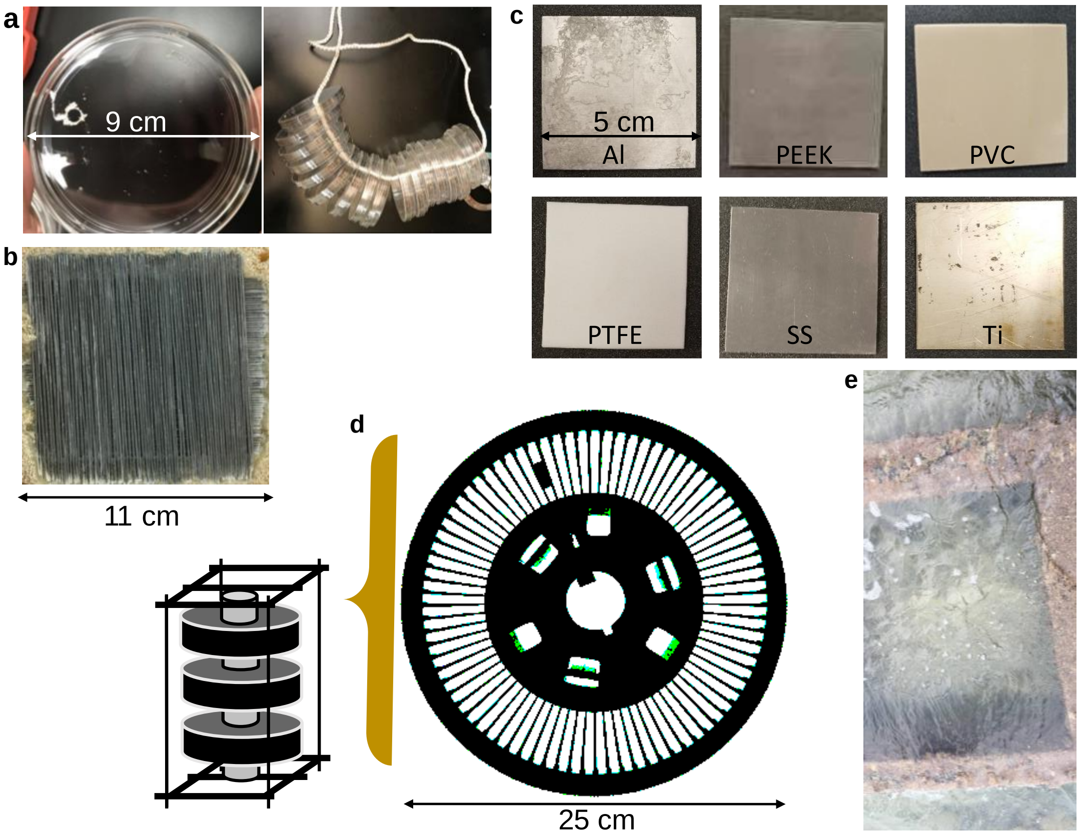

Supplementary Figure 2. Devices and materials used for biofilm development and collection. Biofilms were developed on **a)** Petri dishes, **b)** zinc panels with antifoulant, **c, d)** small panels of man-made materials fixed in a container with matching slots, and **e,** naturally-occurring rocks. Abbreviations: aluminum (Al), poly(ether-ether-ketone) (PEEK), polytetrafluoroethylene (PTFE), poly(vinyl chloride) (PVC), stainless steel (SS) and titanium (Ti).

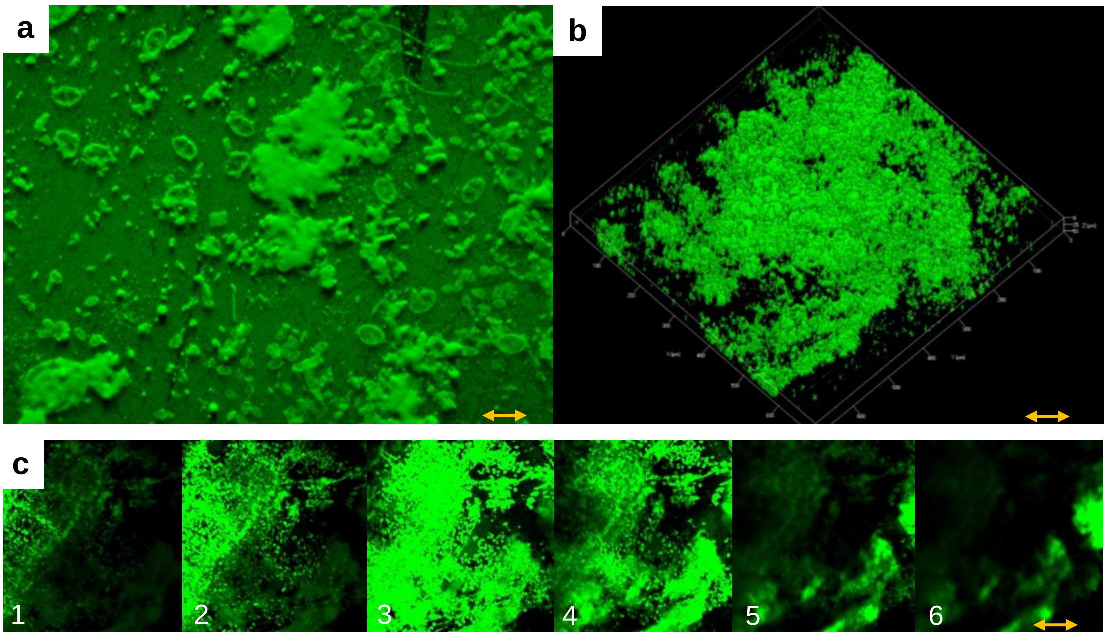

Supplementary Figure 3. Microscopic observation of biofilms under a confocal laser scanning microscope. a) Overall two-dimensional structure. Scale bar: 10 μm. **b)** Overall three-dimensional structure. Scale bar: 100 μm. **c)** Six dissected layers, from the top to the bottom of the biofilm. Scale bar: 25 μm.

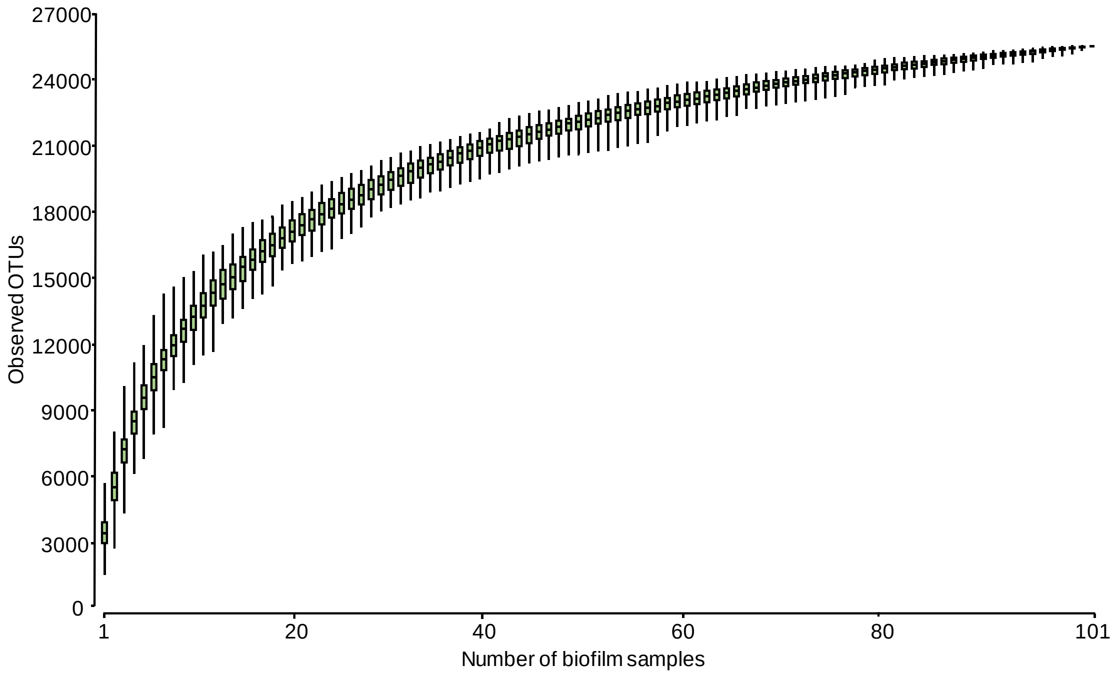

Supplementary Figure 4. Accumulation curve of detected OTUs for 100-fold permuted sampling of the biofilms. Minimal increases towards the end of sampling were observed. Calculations were performed using the 'vegan' package in R with 100 permutations. In a boxplot, central line represents the median, bounds represent upper and lower quartiles, and whiskers represent maximum and minimum.

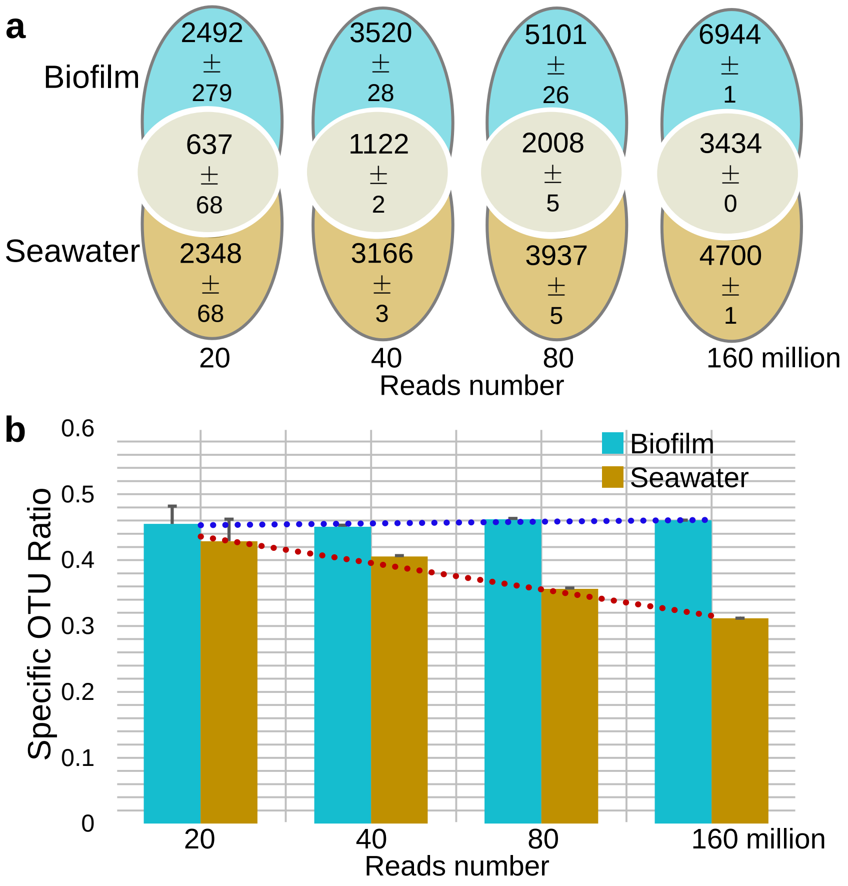

Supplementary Figure 5. Correlation between biofilm or seawater specificity and sequencing depth. A series of numbers (20, 40, 80, and 160 million) of reads were randomly extracted, from one deeply sequenced biofilm metagenome and one seawater metagenome. **a**) The number of OTUs specific to biofilms or seawater. **b**) The ratio of specific OTUs in biofilms or seawater compared to the total number of OTUs in biofilms and seawater. Dot lines indicate the change of the specific OTU ratios along with the increase of sequencing depth. Errors represent standard deviations.

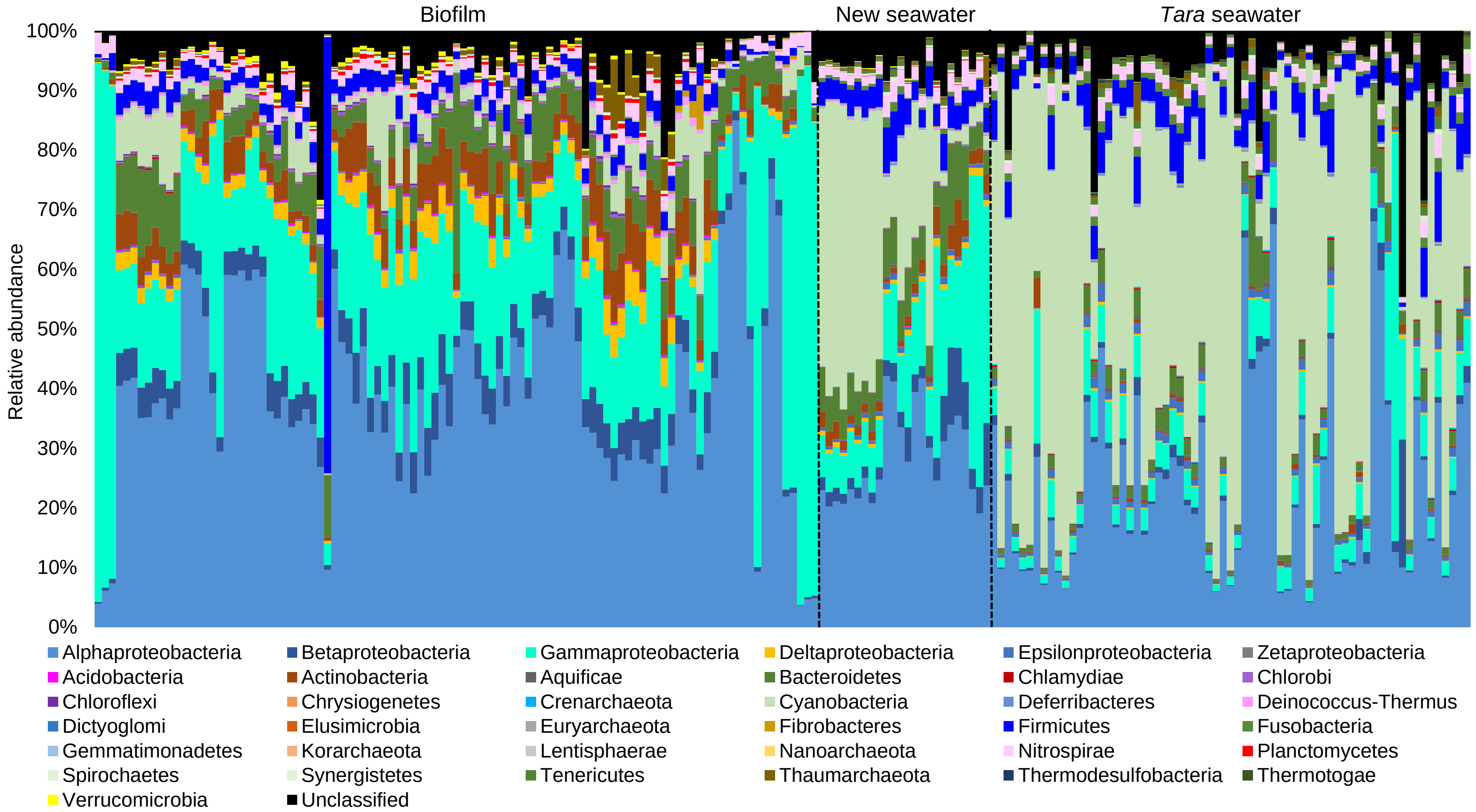

Supplementary Figure 6. Taxonomic composition based on protein-coding marker genes derived from metagenomes. The figure shows the distribution of all phyla across the 101 biofilm and 24 seawater metagenomes that were sequenced as part of the current study and the 67 previously published *Tara* Oceans metagenomes. The order of samples in this figure is the same as that in Supplementary Data 1.

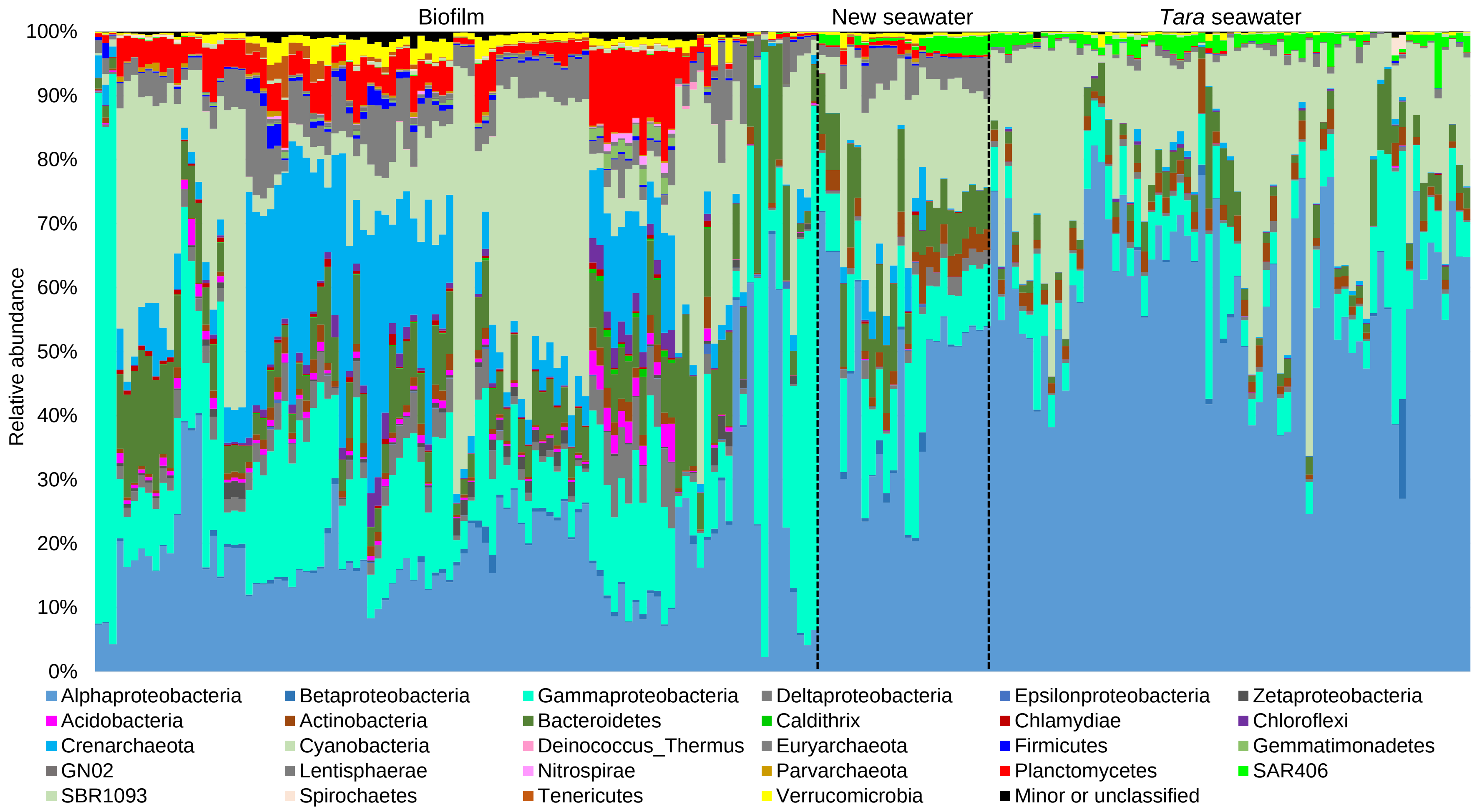

Supplementary Figure 7. Taxonomic composition based on analysis of 16S miTags following gene length and copy number normalization. Abundant phyla (the top 30 phyla in terms of maximum relative abundance) are shown with all other phyla grouped together as 'Minor'. The order of samples in this figure is the same as that in Supplementary Data 1.

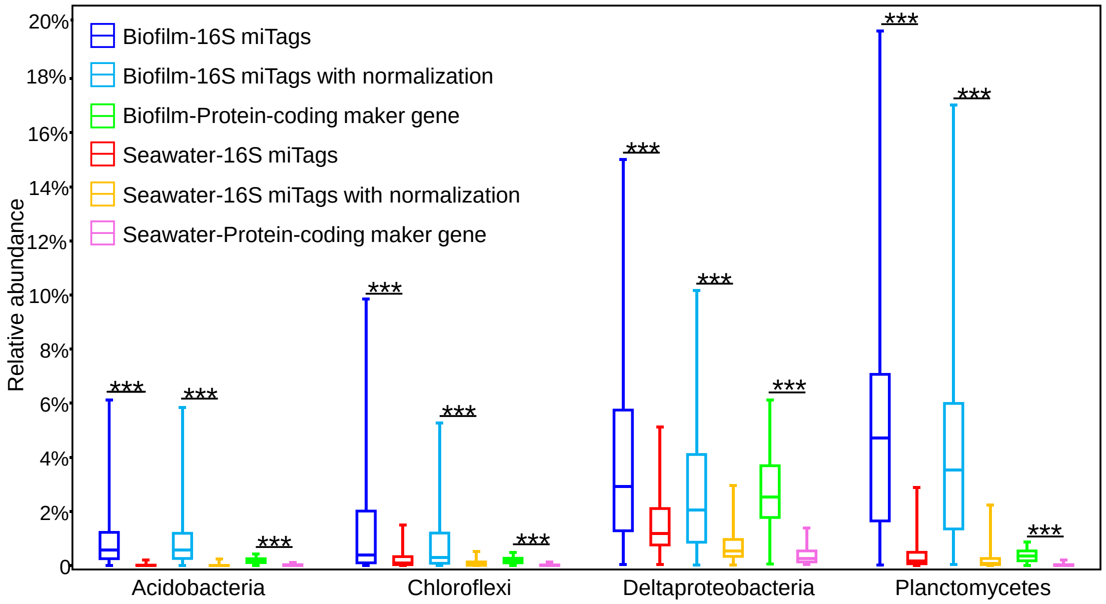

Supplementary Figure 8. Representative microbial phyla differentially enriched in the biofilms (101 biofilms compared with 91 seawater samples). Abundance was calculated by analyzing 16S miTags, 16S miTags normalized by copy number and gene length, and protein-coding marker genes. *** p-value <0.001 (Mann-Whitney U test). In a boxplot, central line represents the median, bounds represent upper and lower quartiles, and whiskers represent maximum and minimum.

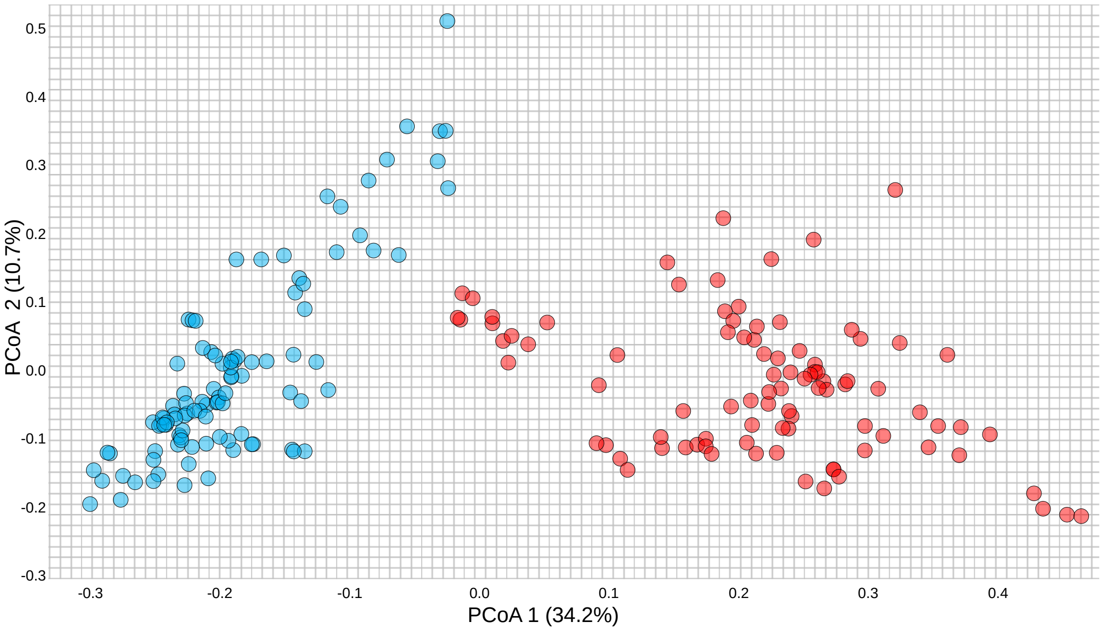

Supplementary Figure 9. **Similarity of the microbial communities based on 16S miTags without data size normalization.** Jaccard distances were calculated based on an OTU matrix derived from 16S miTags without data size normalization, and visualized through PCoA. The blue dots represent biofilms while the red dots represent seawater microbial communities.

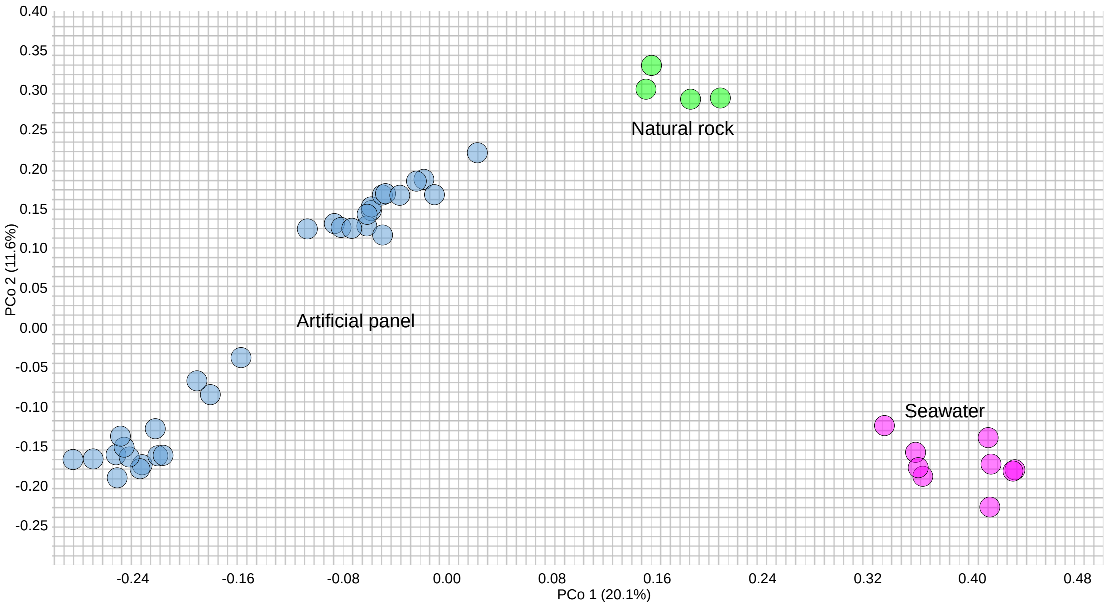

Supplementary Figure 10. **Similarity of the biofilms developed on artificial panels and natural rocks, as well as seawater microbial communities.** All samples included for this analysis were collected in Hong Kong water in 2017. Jaccard similarity derived from the OTU matrix was illustrated by PCoA. These analyses were performed after normalizing the different 16S miTag data to the same library size, i.e. 10,000 sequences per sample.

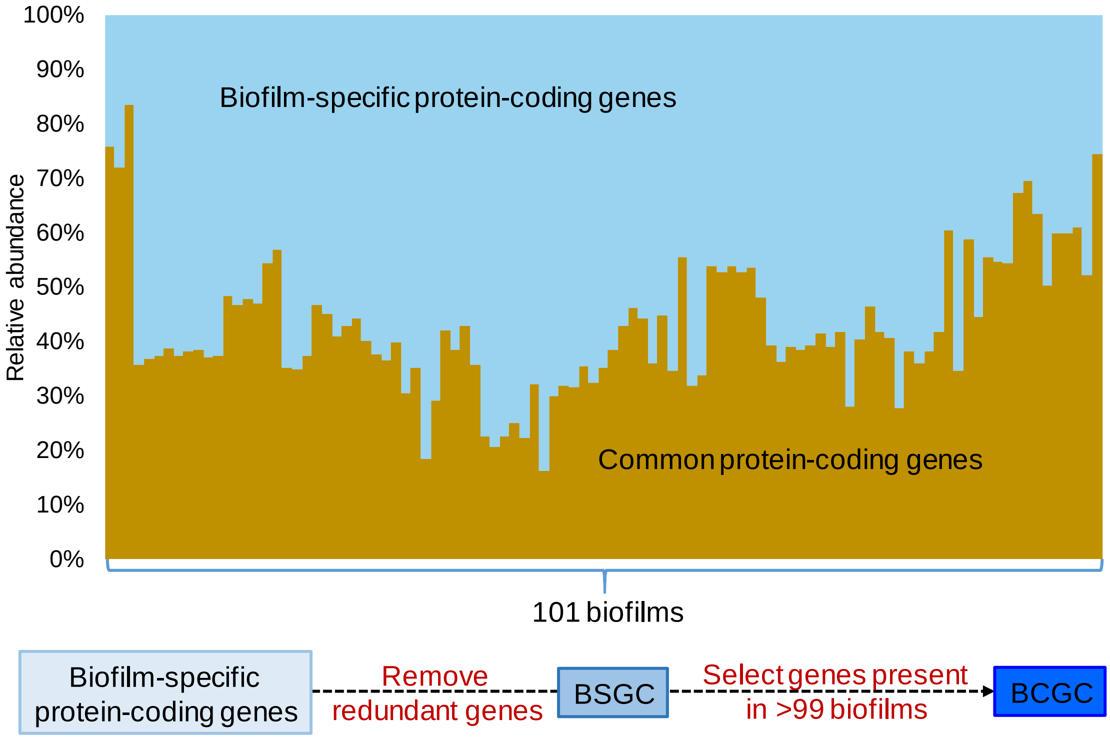

Supplementary Figure 11. Distribution of biofilm-specific protein-coding genes in the 101 biofilm metagenomes. Biofilm-specific protein-coding genes (redundant genes without CD-HIT process) were identified by searching against the OM-RGC and the seawater data from the current study. 'Common protein-coding genes' are genes shared by both biofilm and seawater metagenomes. Biofilm-specific protein-coding genes were subsequently combined and subjected to CD-HIT to generate a nonredundant biofilm-specific gene catalogue (BSGC). The BSGC genes present in more than 99 biofilms are defined as the biofilm core gene catalogue (BCGC).

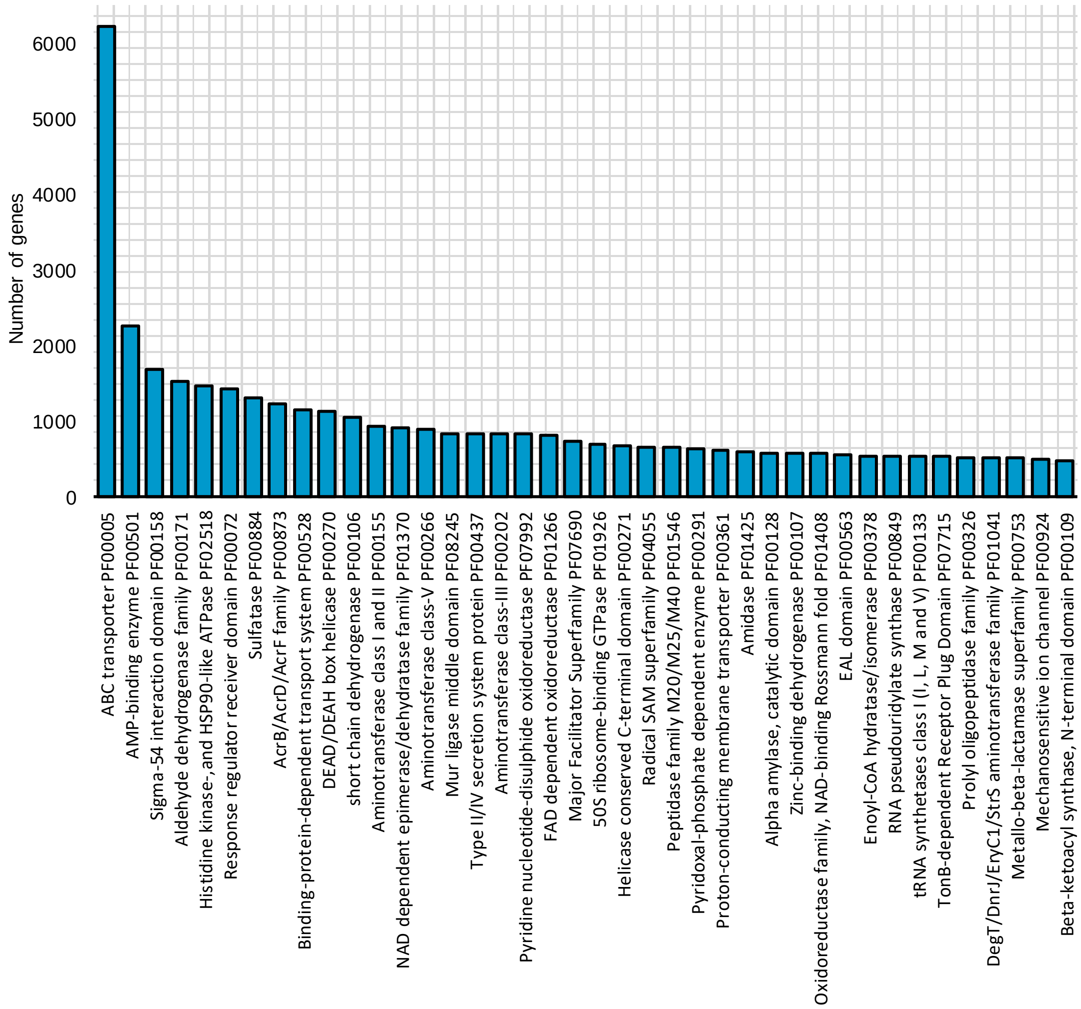

Supplementary Figure 12. The 40 richest Pfam domains in the BCGC. The number of BCGC genes belonging to each Pfam domain is shown.

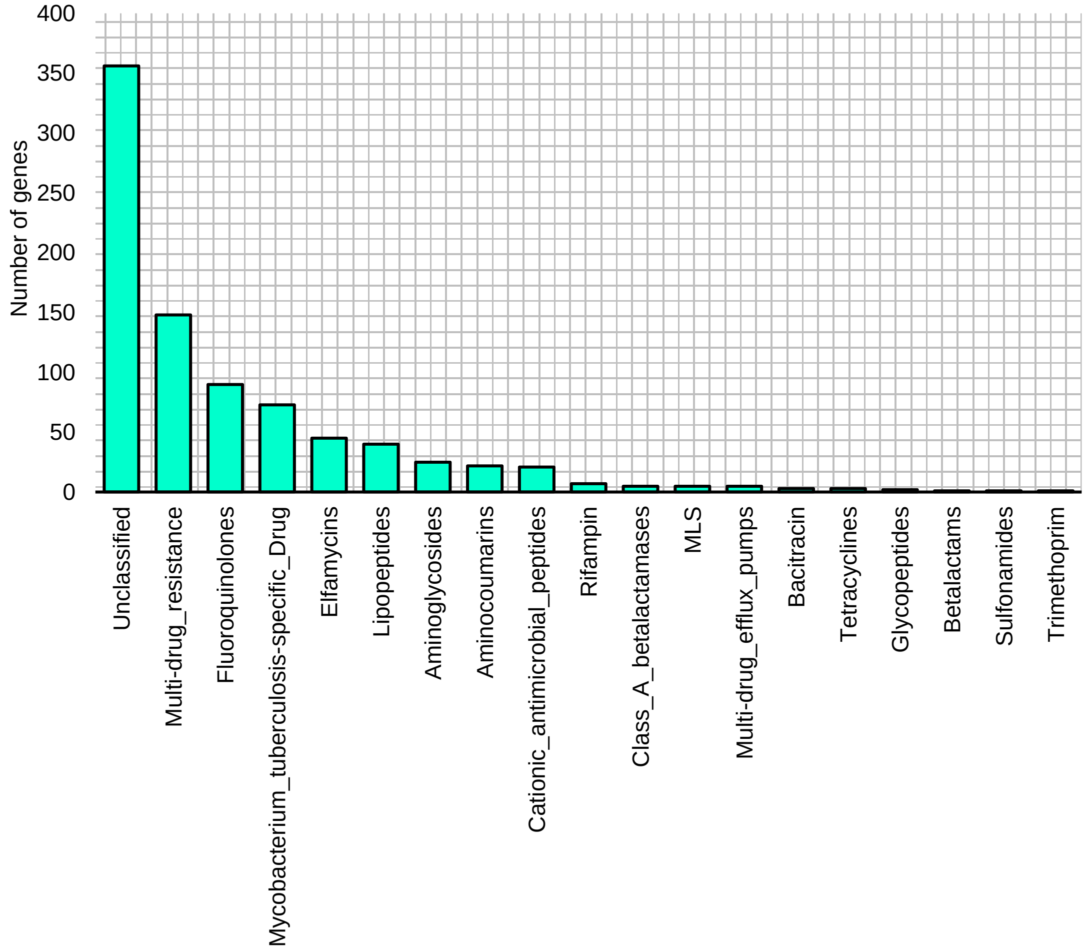

Supplementary Figure 13. Antibiotic resistance genes identified in the BCGC. MLS refers to macrolides, lincosamides, and treptogramins.

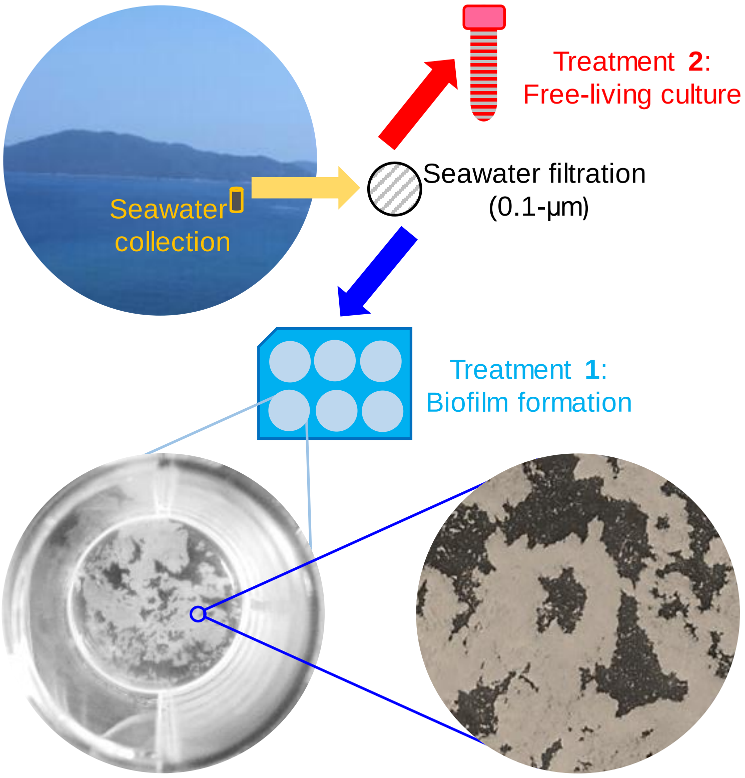

Supplementary Figure 14. Experimental design for the laboratory formation of biofilms and free-living microbial cultures from the same subtidal seawater sample. Each seawater sample collected from the subtidal zone was cultured under two sets of conditions, one allowing for biofilm formation and the other restricting the microorganisms to a free-living state. The experiment was performed in duplicate (two subtidal seawater samples were collected at the initial stage).

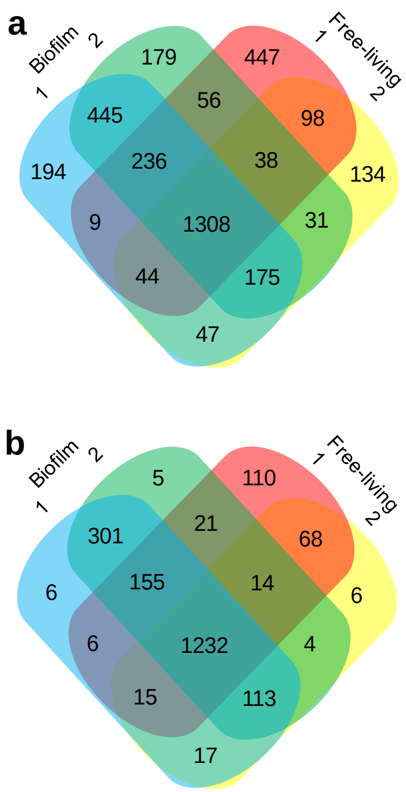

Supplementary Figure 15. Results from the 24 h laboratory experiment testing the proliferation of particular taxa associated with biofilm formation. a) Venn diagram showing the logical relations of all OTUs. **b)** Venn diagram showing abundant OTUs (OTUs with more than nine sequences).

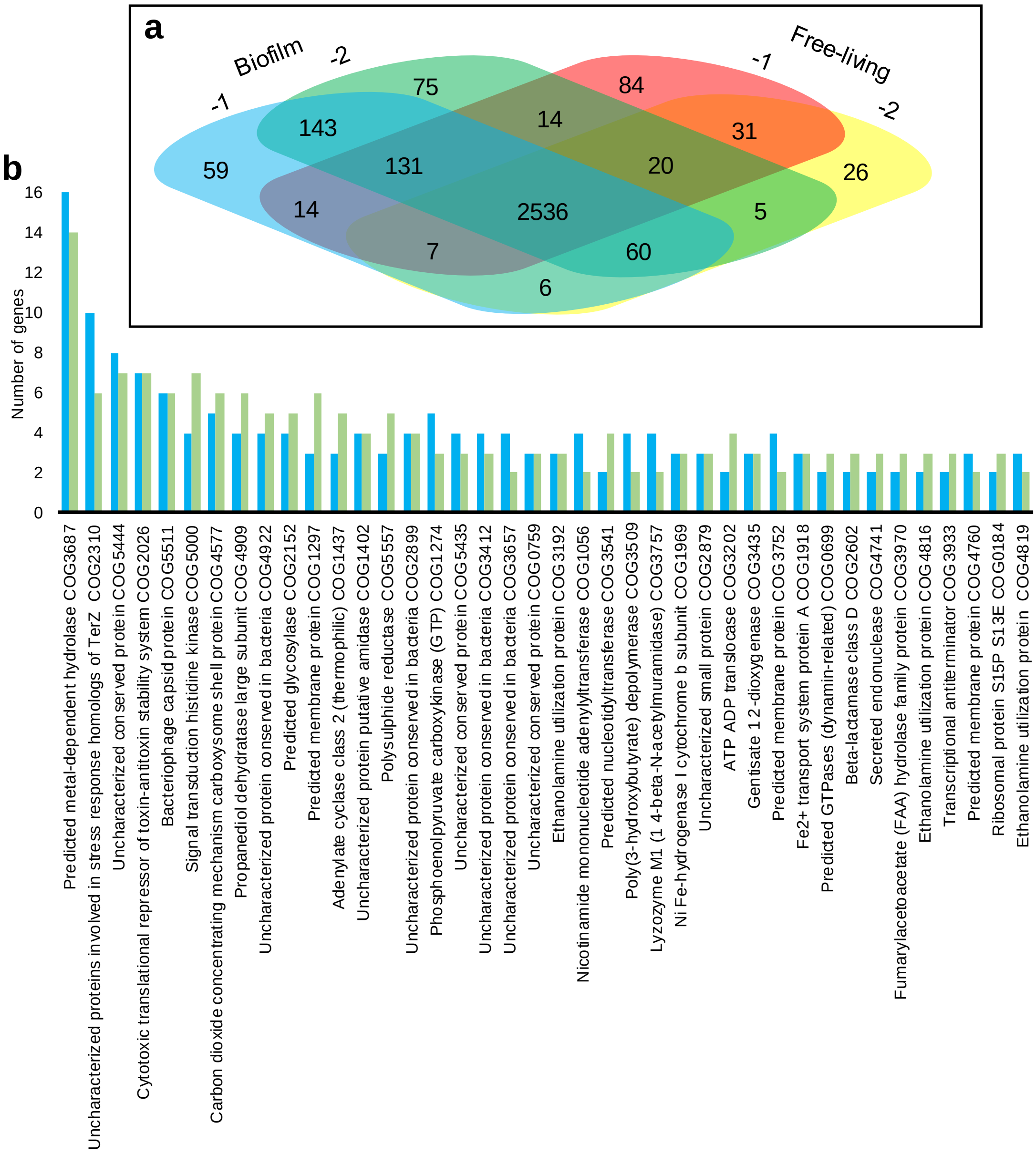

Supplementary Figure 16. Functions associated with biofilm formation in the laboratory. a) Venn diagram showing the logical relations of all COGs. **b)** The 40 most enriched COGs exclusively detected in the two biofilm samples.

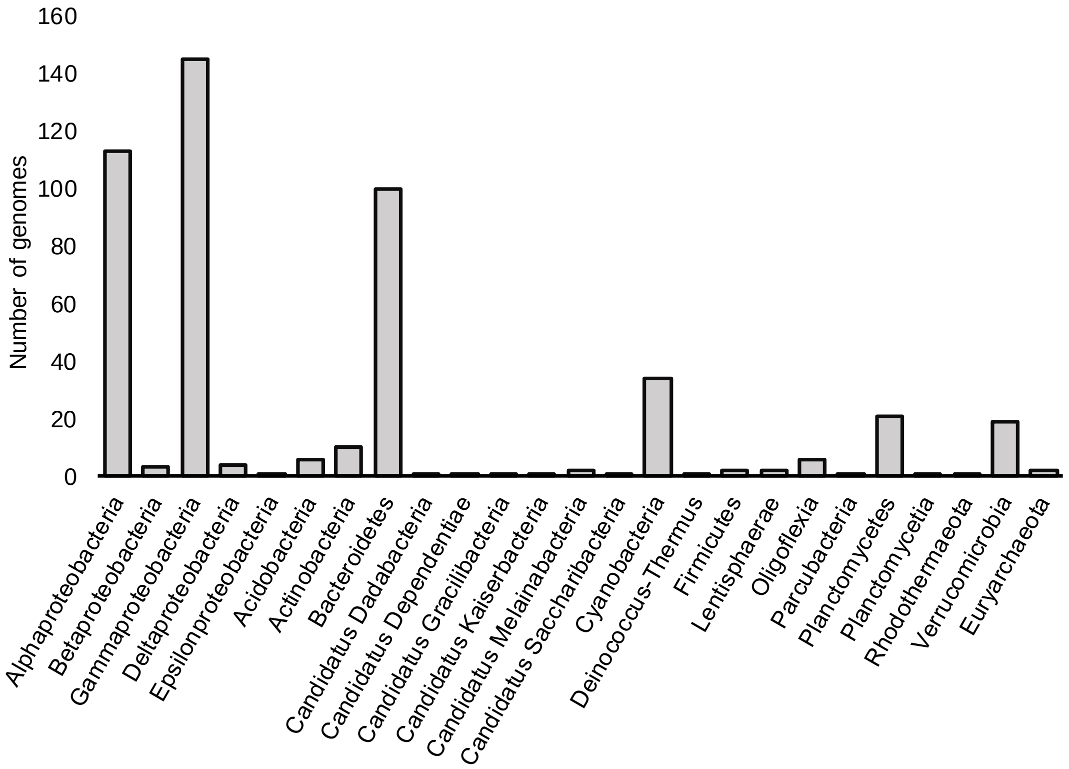

Supplementary Figure 17. Taxonomic affiliation of the 479 microbial genomes recovered from biofilm metagenomes. All contigs used for genome recovery are > 10 Kb.

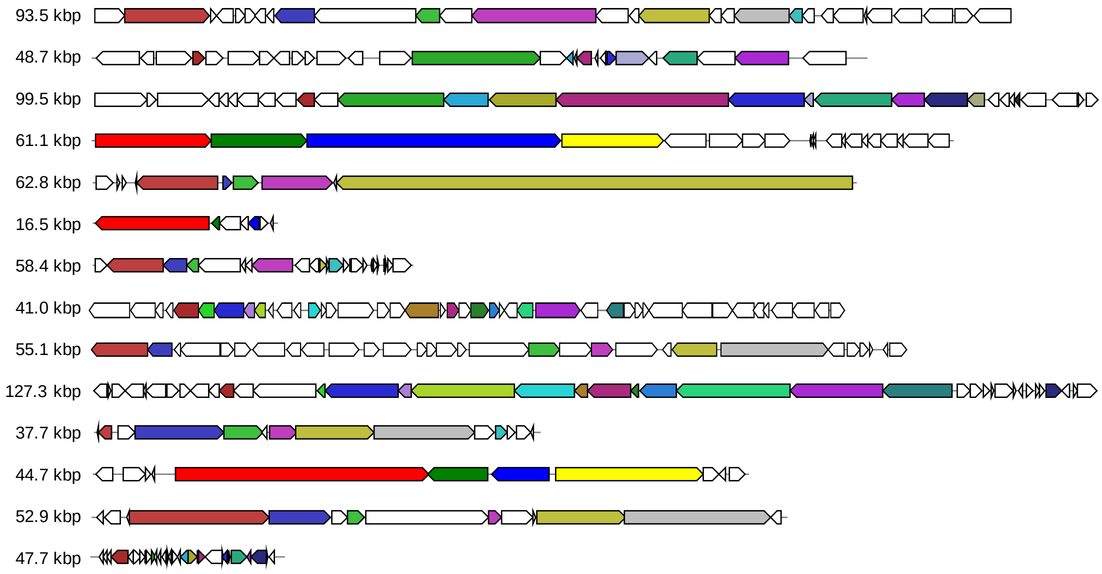

Supplementary Figure 18. PKS and NRPS biosynthetic gene clusters in Acidobacterium sp. BG-534. The longest cluster is 127.3 kb.

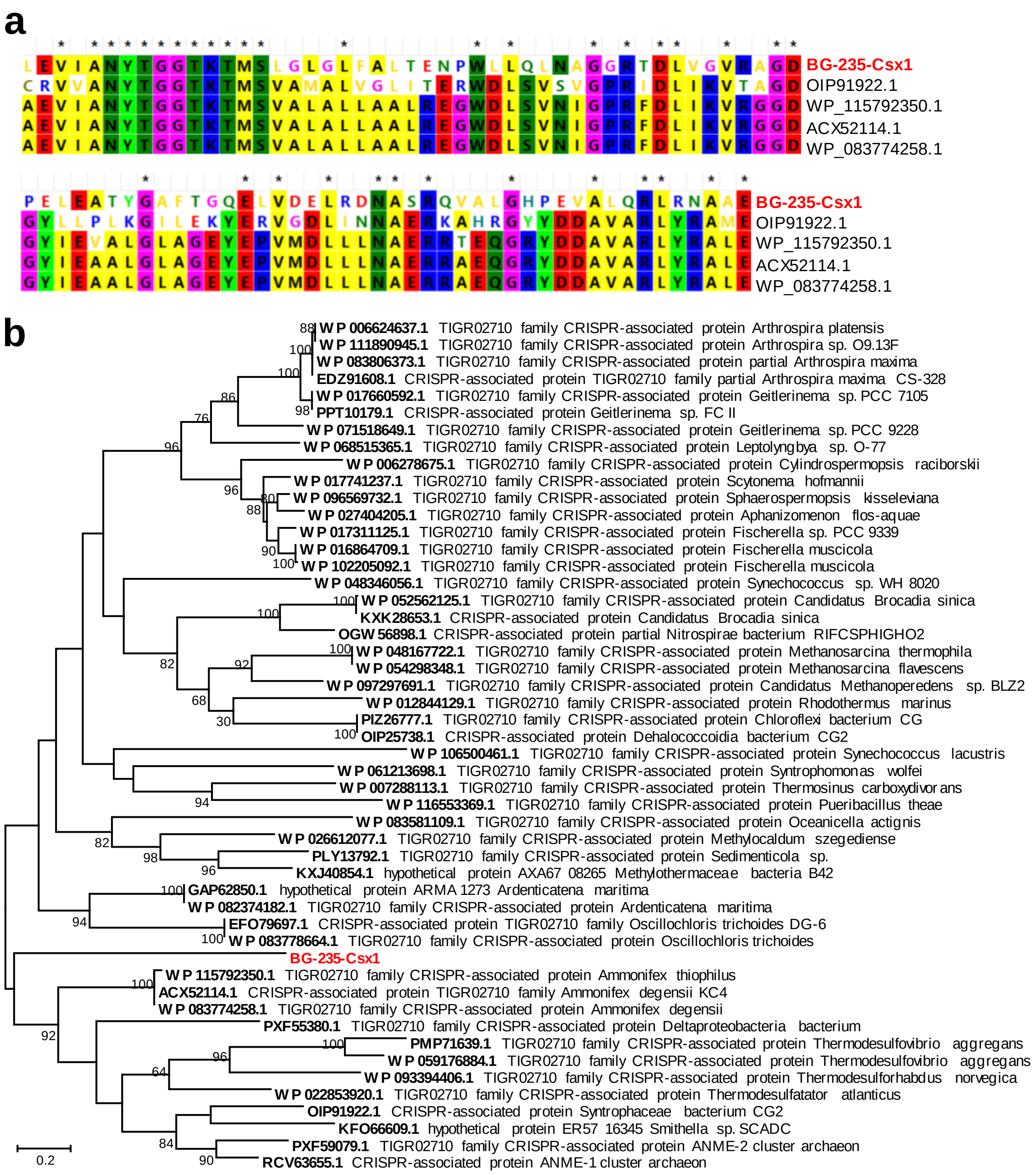

Supplementary Figure 19. Conserved regions and phylogenetic analysis of a *csx1* gene identified from the genome of Acidobacterium sp. BG-235 recovered from the biofilm metagenomes. a) Two conserved regions as revealed by aligning with Csx1 proteins from other bacteria. GenBank numbers: OIP91922.1, TIGR02710 family CRISPR-associated protein [*Syntrophaceae bacterium*]; WP_115792350.1, TIGR02710 family CRISPR-associated protein [*Ammonifex thiophilus*]; TIGR02710 family CRISPR-associated protein [*Ammonifex degensii* KC4]; WP_083774258.1, TIGR02710 family CRISPR-associated protein [*Ammonifex degensii*]. **b)** Phylogenetic analysis of *csx1* using the best hits in the NCBI-Nr database as references. A maximum likelihood tree with 500 replicates is shown.
