# Supplementary material for: Marine biofilms constitute a bank of hidden microbial diversity and functional potential
Source: Nat Commun. 2019 Jan 31;10:517. doi: 10.1038/s41467-019-08463-z (PMC6355793; doi:10.1038/s41467-019-08463-z)
Supplement: Supplementary file 3 — Description of Additional Supplementary Files [file 41467_2019_8463_MOESM3_ESM.pdf]

Description of Additional Supplementary Files

File Name: Supplementary Data 1
Description: Datasets information

File Name: Supplementary Data 2
Description: Summary of the assembled contigs of the 101 biofilm and 24 new seawater Metagenomes

File Name: Supplementary Data 3
Description: List of all OTUs generated by the 16S miTags and used for Venn diagram Comparison

File Name: Supplementary Data 4
Description: Phylum-level composition of the biofilm and seawater microbial communities based on 16S miTags

File Name: Supplementary Data 5
Description: Phylum-level composition of the biofilm and seawater microbial communities based on 16S miTags normalized by copy number and gene length. Relative abundance is given

File Name: Supplementary Data 6
Description: Phylum-level composition of the biofilm and seawater microbial communities based on protein-coding marker genes

File Name: Supplementary Data 7
Description: The OTU table based on rarefied (10,000 miTags per samples) miTags

File Name: Supplementary Data 8
Description: The OTU table (relative abundance) based on total miTags extracted from forward metagenome files of the 101 biofilm and 91 seawater samples

File Name: Supplementary Data 9
Description: The biofilm core functions

File Name: Supplementary Data 10
Description: Antibiotic resistance genes identified from the biofilm core functions

File Name: Supplementary Data 11
Description: The OTUs generated by metagenomic sequencing of laboratory-cultured biofilms and free-living microbes

File Name: Supplementary Data 12
Description: The number of biofilm-specific COGs generated by comparative metagenomic analysis of laboratory-cultured biofilm and free-living microbes

File Name: Supplementary Data 13
Description: Information of the 479 genomes recovered from the biofilm metagenomes

File Name: Supplementary Data 14
Description: Biosynthetic gene clusters identified from genomes

File Name: Supplementary Data 15
Description: CRISPR arrays identified from genomes
