# Supplementary material for: Marine biofilms constitute a bank of hidden microbial diversity and functional potential
Source: Nat Commun. 2019 Jan 31;10:517. doi: 10.1038/s41467-019-08463-z (PMC6355793; doi:10.1038/s41467-019-08463-z)
Supplement: Supplementary file 19 — Reporting Summary [file 41467_2019_8463_MOESM19_ESM.pdf]

nature research

Corresponding author(s): Pei-Yuan Qian

Reporting Summary

Nature Research wishes to improve the reproducibility of the work that we publish. This form provides structure for consistency and transparency in reporting. For further information on Nature Research policies, see Authors & Referees and the Editorial Policy Checklist.

Statistical parameters

When statistical analyses are reported, confirm that the following items are present in the relevant location (e.g. figure legend, table legend, main text, or Methods section).

n/a	Confirmed	
☐	☒	The exact sample size (*n*) for each experimental group/condition, given as a discrete number and unit of measurement
☐	☒	An indication of whether measurements were taken from distinct samples or whether the same sample was measured repeatedly
☐	☒	The statistical test(s) used AND whether they are one- or two-sided<br>*Only common tests should be described solely by name; describe more complex techniques in the Methods section.*
☒	☐	A description of all covariates tested
☒	☐	A description of any assumptions or corrections, such as tests of normality and adjustment for multiple comparisons
☒	☐	A full description of the statistics including central tendency (e.g. means) or other basic estimates (e.g. regression coefficient) AND variation (e.g. standard deviation) or associated estimates of uncertainty (e.g. confidence intervals)
☒	☐	For null hypothesis testing, the test statistic (e.g. *F*, *t*, *r*) with confidence intervals, effect sizes, degrees of freedom and *P* value noted<br>*Give P values as exact values whenever suitable.*
☒	☐	For Bayesian analysis, information on the choice of priors and Markov chain Monte Carlo settings
☒	☐	For hierarchical and complex designs, identification of the appropriate level for tests and full reporting of outcomes
☒	☐	Estimates of effect sizes (e.g. Cohen's *d*, Pearson's *r*), indicating how they were calculated
☒	☐	Clearly defined error bars<br>*State explicitly what error bars represent (e.g. SD, SE, CI)*

Our web collection on statistics for biologists may be useful.

Software and code

Policy information about availability of computer code

Data collection	Data were collected by sampling from marine environments and DNA sequencing on the HiSeq sequencing Systems.
Data analysis	All the software and scripts used in this manuscript are publicly available or published in previous studies. All the related references are listed after the software in the Method part of the main text. The software used in this manuscript includes NGS QC Toolkit (version 2.0), MEGAHIT (version 1.0.2), HMMER (version 3.1), Bowtie2, Parallel-META3, QIIME (version 1.91), MetaGeneMark (version 2.8), MEGA7, MetaBAT1, ChechM1, antiSMASH 4.0.

For manuscripts utilizing custom algorithms or software that are central to the research but not yet described in published literature, software must be made available to editors/reviewers upon request. We strongly encourage code deposition in a community repository (e.g. GitHub). See the Nature Research guidelines for submitting code & software for further information.

Data

Policy information about availability of data

All manuscripts must include a data availability statement. This statement should provide the following information, where applicable:

- Accession codes, unique identifiers, or web links for publicly available datasets
- A list of figures that have associated raw data
- A description of any restrictions on data availability

All metagenomic datasets have been deposited in the NCBI database under BioProject accession no. PRJNA438384 (BioSample no. SAMN08714533 for the 101 biofilm metagenomes; BioSample no. SAMN0871453 for the 24 adjacent seawater metagenomes; BioSample no. SAMN08714627 for the two biofilm and two seawater metagenomes that have been sequenced for more than 110 Gb data; and BioSample no. SAMN08714628 for the metagenomes of the laboratory cultured biofilm and free-living microbes). Gene catalogues, including the biofilm specific genes and core genes, as well as the seawater gene sequences are uploaded to figshare (BSGC, https://figshare.com/s/7e193dceb329c02cb166, DOI: 10.6084/m9.figshare.6743327; BCGC, https://figshare.com/s/53f27d05d443c6c947e5, DOI: 10.6084/m9.figshare.6743324; NSGC, https://figshare.com/s/7815ea074e5c2d7ef1c5, DOI: 10.6084/m9.figshare.6743459). The 479 microbial genomes are uploaded to figshare (https://figshare.com/s/2994fdafe79112b99907, DOI: 10.6084/m9.figshare.7082684). Supplementary Figs. 1 to 19, Supplementary Data 1 to 15 are present in the supplementary materials.

Field-specific reporting

Please select the best fit for your research. If you are not sure, read the appropriate sections before making your selection.

☒ Life sciences ☐ Behavioural & social sciences ☐ Ecological, evolutionary & environmental sciences

For a reference copy of the document with all sections, see nature.com/authors/policies/ReportingSummary-flat.pdf

Life sciences study design

All studies must disclose on these points even when the disclosure is negative.

Sample size	101 biofilm samples and 24 seawater samples were included for analyses.
Data exclusions	No data were excluded.
Replication	The related files, including intermediate results required to reproduce the results presented in the manuscript, are provided in the Data availability part of the manuscript.
Randomization	Not applicable.
Blinding	Not applicable.

Reporting for specific materials, systems and methods

Materials & experimental systems

n/a	Involved in the study
☒	☐ Unique biological materials
☒	☐ Antibodies
☒	☐ Eukaryotic cell lines
☒	☐ Palaeontology
☒	☐ Animals and other organisms
☒	☐ Human research participants

Methods

n/a	Involved in the study
☒	☐ ChIP-seq
☒	☐ Flow cytometry
☒	☐ MRI-based neuroimaging
